# Association of spinopelvic mobility and osteosarcopenia with total hip arthroplasty outcomes

**DOI:** 10.1002/jeo2.70395

**Published:** 2025-08-05

**Authors:** Yoshinori Okamoto, Hitoshi Wakama, Takafumi Saika, Kengo Tani, Shuhei Otsuki

**Affiliations:** ^1^ Department of Orthopedic Surgery Osaka Medical and Pharmaceutical University Takatsuki Japan; ^2^ Department of Orthopedic Surgery Saiseikai Ibaraki Hospital Ibaraki Japan

**Keywords:** nutrition, osteosarcopenia, patient‐reported outcomes, psoas, sacral slope, spinopelvic mobility, total hip arthroplasty

## Abstract

**Purpose:**

This study assessed the association of spinopelvic mobility and osteosarcopenia with the achievement of a patient‐acceptable symptom state in patient‐reported outcomes after total hip arthroplasty.

**Methods:**

This retrospective study included 244 patients who underwent primary total hip arthroplasty with a minimum follow‐up of 24 months. Spinopelvic mobility was assessed using changes in sacral slope during postural transitions, classified as stiff (<10°), normal (10°–30°), or hypermobility (≥30°). Osteosarcopenia was defined by psoas muscle area on computed tomography and lumbar bone mineral density. Outcomes were assessed using the EuroQol 5‐Dimension and the Hip Disability and Osteoarthritis Outcome Score–Joint Replacement. Multivariate logistic regression analysis was performed to identify predictors of a patient‐acceptable symptom state achievement on the EuroQol 5‐Dimension. Propensity score matching yielded 35 patients with limited mobility and 70 controls.

**Results:**

Physiological spinopelvic mobility (odds ratio 0.66, 95% confidence interval 0.56–0.78, *p* = 0.028) and the absence of osteosarcopenia (odds ratio 0.68, 95% confidence interval 0.49–0.94, *p* = 0.031), along with older age (odds ratio 1.12, 95% confidence interval 1.01–1.24, *p* = 0.046), were associated with higher patient‐acceptable symptom state achievement rates for EuroQol 5‐Dimension. Propensity‐matched analysis revealed that patients with limited mobility exhibited significantly lower nutritional indices (*p* = 0.008), EuroQol 5‐Dimension (*p* < 0.001), Hip Disability and Osteoarthritis Outcome Score–Joint Replacement (*p* < 0.001), and satisfaction scores (*p* = 0.005). Dynamic sacral slope changes correlated significantly with nutritional and muscle indices (*p* < 0.001).

**Conclusion:**

Preoperative spinopelvic stiffness and osteosarcopenia independently predict poor functional recovery following total hip arthroplasty. This indicates the need for comprehensive preoperative assessments addressing both spinopelvic biomechanics and musculoskeletal health to optimise total hip arthroplasty strategies. Future research should explore tailored surgical approaches to improve outcomes in vulnerable populations, particularly those with impaired spinopelvic mobility and osteosarcopenia.

**Level of Evidence:**

Level III, retrospective cohort study.

**Abbreviations**
CI: confidence interval
CT: computed tomography
EQ‐5D: EuroQol 5‐Dimension 3‐Level scale
HOOS‐JR: Hip Disability and Osteoarthritis Outcome Score–Joint Replacement
MCID: minimal clinically important difference
PASS: patient‐acceptable symptom state
PMA: psoas muscle area
PNI: prognostic nutritional index
SS: sacral slope
THA: total hip arthroplasty

## INTRODUCTION

Spinopelvic mobility plays a crucial role in acetabular component positioning, joint stability, and functional recovery following total hip arthroplasty (THA) [1, 11, 12, 23]. The dynamic interaction between the pelvis and spine during postural transitions, particularly between standing and sitting, influences hip biomechanics and patient‐reported outcomes [12, 21]. Restricted spinopelvic mobility alters acetabular orientation and may contribute to impingement, dislocation, and persistent pain [11, 23]. As a result, preoperative spinopelvic assessment is increasingly emphasised in THA planning [18, 21]. However, while abnormal spinopelvic mobility is associated with mechanical complications, its direct impact on postoperative recovery and patient satisfaction remains poorly understood.

In parallel, osteosarcopenia, characterised by low bone density and muscle mass, is an emerging concern in ageing populations [13, 17, 19]. This condition has been linked to higher fall risk, impaired rehabilitation capacity, and reduced functional independence, which may negatively influence THA outcomes [17]. Despite its growing clinical recognition, how osteosarcopenia specifically affects postoperative recovery following THA remains underexplored.

While previous studies have examined spinopelvic alignment and osteosarcopenia independently, their potential interaction and additive effects on THA outcomes remain largely unknown [10, 16]. Spinopelvic stiffness may increase biomechanical stress on the hip and spine, while osteosarcopenia could further reduce adaptive capacity, potentially leading to compromised functional recovery [1, 5]. Understanding these interactions is critical for optimising preoperative risk stratification and surgical decision‐making [12, 23]. Recent reports have highlighted key biomechanical considerations in THA, such as implant design, lumbopelvic alignment, and pelvic obliquity [7, 10, 16]. This study aimed to evaluate the independent and combined effects of preoperative spinopelvic stiffness and osteosarcopenia on functional recovery following THA. We hypothesised that both spinopelvic stiffness and osteosarcopenia would be independently associated with inferior patient‐reported outcomes following THA. By simultaneously assessing these biomechanical and musculoskeletal factors, this study provides novel insights into their interplay and clinical implications for preoperative risk stratification and postoperative management strategies.

## MATERIALS AND METHODS

### Ethical considerations

This study was approved by the institutional ethics review board and was conducted in accordance with the Declaration of Helsinki. Written informed consent for surgery and data use in research was obtained from all participants.

### Study design and population

**Figure 1:**
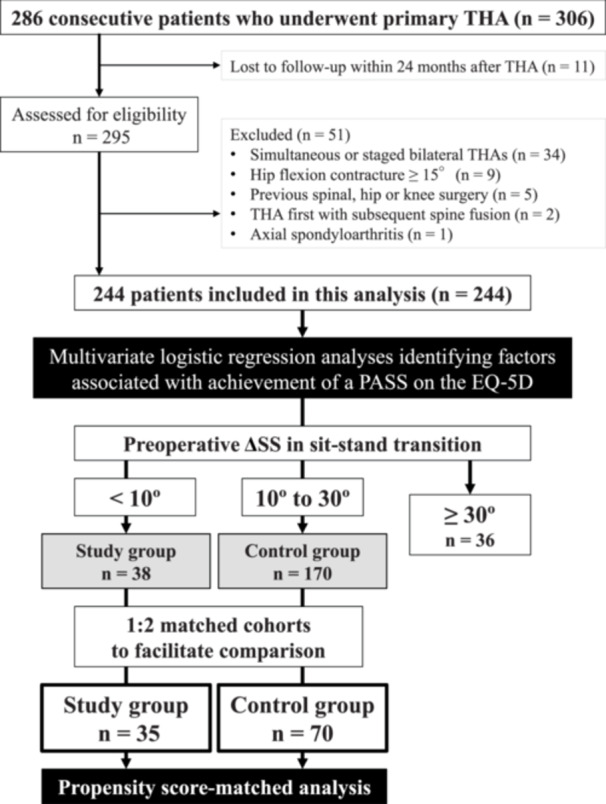
Flowchart of patient selection, based on predefined inclusion and exclusion criteria. EQ‐5D, European Quality of Life 5‐Dimension 3‐Level; PASS, patient‐acceptable symptom state; ΔSS, change in sacral slope; THA, total hip arthroplasty.

This single‐centre, retrospective cohort study analysed THA outcomes in 286 consecutive patients who underwent unilateral primary THA between 1 September 2018 and 31 March 2023. To ensure homogeneity in the study population and minimise potential confounders, only patients of Asian descent were included. This was determined based on self‐report at the time of admission and confirmed through medical records, including the national health insurance registry and documented family origin. Of the 295 eligible THAs performed during the study period with available 24‐month follow‐up data, 51 were excluded, primarily due to simultaneous or staged bilateral THAs (34 hips, 67%). The final analysis included 244 hips from 244 patients (Figure 1). There were no significant differences in baseline demographics and clinical characteristics between included and excluded patients (*p* > 0.05). The mean follow‐up duration was 40 months (range, 24–72 months).

### Surgical procedure and postoperative protocol

All THAs were conducted by four experienced arthroplasty surgeons who used a direct lateral approach with the patient in the lateral decubitus position [18, 19]. The surgeries were performed using exclusively cemented prostheses in accordance with institutional protocols favouring cemented fixation for older adults or patients with osteoporosis based on previously established techniques [19]. The acetabular components consisted of ultra‐high‐molecular‐weight cross‐linked polythene liners (K‐MAX CLPE cup for 203 hips and EXL GP Socket for 35 hips, both from Kyocera Medical Corporation, Osaka, Japan) or dual mobility systems (Avantage Acetabular System for 6 hips; Zimmer‐Biomet, Warsaw, IN). The femoral components included cobalt–chromium heads and stems, such as the K‐MAX SC hip system (214 hips; Kyocera Medical Corporation), Exeter Universal femoral stems (25 hips; Stryker Corporation, Mahwah, NJ), and CPT 12/14 hip stems (5 hips; Zimmer‐Biomet). The use of dual mobility systems was limited to six patients (2.5%) and was determined by the operating surgeon based on patient‐specific factors. Given the small number, these cases were unlikely to significantly influence the primary outcomes. Postoperatively, all patients followed a standardised rehabilitation protocol with full weight‐bearing starting on postoperative Day 1 under the supervision of physical therapists. The rehabilitation programme emphasised gait training, pain management, and progressive functional recovery.

### Assessment methods

The primary endpoints were the European Quality of Life 5‐Dimension 3‐Level (EQ‐5D) scale and the visual analogue scale for hip and low back pain, used to assess functional and pain‐related outcomes [3, 6]. The EQ‐5D was a standardised health‐related quality‐of‐life measure assessing five dimensions: mobility, pain/discomfort, anxiety/depression, self‐care and daily activities [6]. Pain severity was measured on a 100‐mm visual analogue scale, where “0” indicated no pain and “100” represented the worst imaginable pain [3]. Assessments were performed preoperatively, at 2 weeks, 6 and 12 months postoperatively, and annually thereafter, with final assessments at the last follow‐up visit. Secondary outcomes included the Hip Disability and Osteoarthritis Outcome Score–Joint Replacement (HOOS‐JR), a six‐item measure assessing symptoms, pain, and functional limitations, with scores converted to a 0–100 scale (higher scores indicating better function) [14]. HOOS‐JR measurements were collected preoperatively and at final follow‐up. Surgical success was evaluated based on the minimal clinically important difference (MCID) and the patient‐acceptable symptom state (PASS), both of which were adopted from previously published anchor‐based studies [3, 4, 6, 14]. The MCID represented the smallest change in a score that patients perceive as beneficial, while the PASS reflected the threshold beyond which patients consider their symptom state satisfactory. These thresholds were derived using global satisfaction ratings or symptom state anchors in THA populations. For EQ‐5D and HOOS‐JR, the established PASS values were > 0.77 and > 70 points, respectively [6, 14]. These metrics were increasingly utilised to assess treatment outcomes and patient satisfaction [4, 14]. Lastly, patient satisfaction was assessed using a custom questionnaire that included domains such as pain relief, the ability to perform daily and recreational activities, and overall satisfaction, which was rated on a 5‐point Likert scale [8]. Responses were scored on a 5‐point scale, ranging from ‘very dissatisfied’ to ‘very satisfied’, with an aggregate satisfaction score calculated by using equal weighting.

Nutritional status was assessed using the Prognostic Nutritional Index (PNI), which integrates serum albumin and total lymphocyte count to reflect protein reserves and immune function. It was calculated as:

PNI=(10×serumalbumin[g/dL])+(0.005×totallymphocytecount[/mm³]).

This index has been validated as a prognostic marker in surgical and orthopaedic patients, with lower values (<40) associated with poor outcomes, including delayed recovery and increased complication rates [2, 15].

### Radiographic assessment

**Figure 2:**
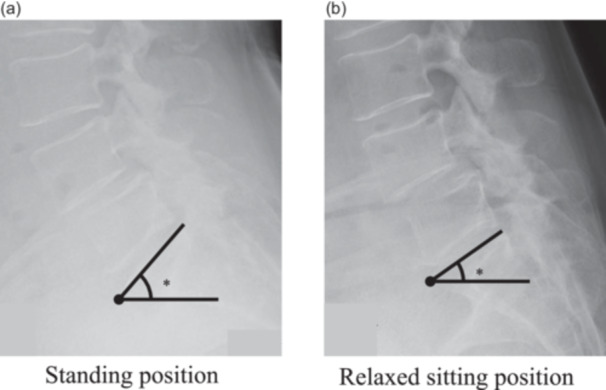
Radiographic evaluation of sacral slope in two postures, obtained preoperatively and at final follow‐up. (a) Evaluation in a natural standing position. (b) Evaluation in a relaxed sitting position. Sacral slope (*) is measured as the angle between the superior endplate of S1 and a horizontal reference line (solid lines).

Spinopelvic alignment was assessed preoperatively and at the last follow‐up (minimum 24 months postoperatively) using lateral spinal radiographs extending from C7 to the pelvis. For standing radiographs, patients stood naturally with their gaze directed forward, hands resting on a support, and upper limbs relaxed. For sitting radiographs, patients sat on a height‐adjustable stool with their femur parallel to the floor and feet flat on the ground, ensuring a neutral pelvic position. Sacral slope (SS) was measured as the angle between the superior endplate of the S1 vertebra and a horizontal reference line, while the change in SS (ΔSS) was calculated as the difference between the standing and sitting SS values, reflecting spinopelvic mobility (Figure 2) [1, 12]. Normal spinopelvic mobility was generally defined as ΔSS between 10° and 30°. A ΔSS < 10° was classified as spinopelvic stiffness, reflecting reduced pelvic adaptability and potential compensatory stress on adjacent joints. Conversely, ΔSS > 30° indicated hypermobility, which may predispose patients to instability and altered joint loading patterns [9, 21]. These thresholds were essential for assessing spinopelvic biomechanics, particularly for THA, as they influence implant positioning, stability, and the risk of complications, such as dislocation [11, 12, 21, 23]. A ΔSS of <10° has been previously identified as a threshold indicating spinopelvic stiffness, associated with decreased pelvic adaptability and a higher risk of impingement, prosthetic instability, and suboptimal functional recovery [12, 17]. Given these findings, this clinically meaningful threshold was adopted to stratify patients based on spinopelvic mobility. Moreover, recent biomechanical analyses have suggested that limited spinopelvic mobility below this threshold altered functional acetabular orientation, potentially affecting hip joint loading patterns and postural stability after THA [23]. Accordingly, the 10° threshold serves as a key criterion for identifying patients at risk for suboptimal postoperative recovery [11, 12, 21, 23].

### Preoperative bone and muscle quality assessment

Preoperative assessments were performed using computed tomography (CT; Aquilion Prime SP; Canon Medical Systems, Otawara, Japan) and analysed via Picture Archiving and Communication Systems (IMPAX; Agfa Healthcare, Mortsel, Belgium). Standard CT parameters (120 kV, 375 mA, 1.0‐mm slice thickness) were used. The mean interval between imaging and surgery was 44 days (1–51 days). As part of standard preoperative planning, CT scans were routinely obtained for all patients and were not performed specifically for research purposes.

Osteosarcopenia was defined based on CT‐derived psoas muscle area (PMA) and bone density, using previously validated thresholds established in orthopaedic and geriatric populations [17, 20, 22]. Dual‐energy X‐ray absorptiometry scans of the lumbar spine were performed using a Hologic Horizon Wi (Hologic, Marlborough, MA, USA). Measurements were obtained at four vertebral levels (L1–L4) with the patient in a supine position. To reduce lumbar lordosis, a manufacturer‐provided support block was placed under the calves, elevating the legs during the scan. The vertebral levels were identified by drawing an inter critical line approximately corresponding to the L4/L5 level. Osteoporosis was diagnosed according to the World Health Organization criteria: T‐score < −2.5 defined osteoporosis, −2.5 ≤ T‐score < −1.0 indicated osteopenia, and T‐score ≥ −1.0 was considered normal. The T‐scores and bone mineral density of the L1 vertebra were recorded for each patient [20].

**Figure 3:**
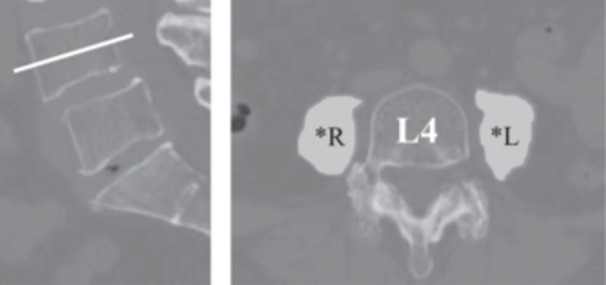
Cross‐sectional area of the right (*R) and left psoas major (*L) at the mid‐level of the L4 vertebral body, determined by manual tracing of preoperative computed tomography images. The sum of these areas represents the total psoas muscle area, which correlates positively with total skeletal muscle volume, serving as a prognostic indicator for sarcopenia.

The cross‐sectional PMA was measured at the L4 vertebral level using axial CT images, as this level, identified using sagittal multiplanar reconstruction of CT images, has been shown to correlate with whole‐body skeletal muscle mass (Figure 3) [22]. Low PMA was defined as <2200 mm² for men and <1200 mm² for women [5, 22]. To assess inter‐ and intra‐rater reliability, 10 hips were randomly selected for PMA by two orthopaedic surgeons at an average interval of 4.2 weeks. The intra‐ and interclass correlation coefficients were 0.816 and 0.835, indicating high measurement reliability. The intra‐ and inter‐rater agreements were 93% and 87% (discrepancy < 200 mm²), respectively.

### Statistical analysis

Patients were categorised based on whether they achieved the PASS threshold on the EQ‐5D (>0.77) at the final follow‐up [6]. Continuous variables were compared between these groups using the Mann–Whitney *U* test for non‐normally distributed data, while categorical variables were analysed using Pearson's chi‐square or Fisher's exact tests, depending on expected cell counts. Normality was assessed using the Shapiro–Wilk test. To identify independent predictors of PASS achievement, multivariate logistic regression analysis was performed, adjusting for age, sex, body mass index, Charlson Comorbidity Index, osteosarcopenia, pelvic incidence, and spinopelvic mobility (≥10°) [12, 13]. Multicollinearity was assessed using the variance inflation factor, which quantifies the degree of correlation among independent variables in a regression model. Only variables with a variance inflation factor below 10 were included in the final model to ensure stable coefficient estimates and minimise redundancy. To assess whether spinopelvic mobility and osteosarcopenia exerted a combined effect on outcomes, an interaction term (spinopelvic mobility × osteosarcopenia) was included in the regression model. To minimise confounding, propensity score matching was performed using a 1:2 nearest‐neighbour matching algorithm with a calliper width of 0.20. Matching variables included age, sex, body mass index, and Charlson Comorbidity Index. Standardised mean differences < 0.10 were considered indicative of adequate covariate balance between matched groups. Subgroup analyses were performed by stratifying patients according to preoperative spinopelvic mobility. Patients with ΔSS < 10° were defined as the study group, and those with ΔSS ≥ 10° were assigned to the control group to examine differences in postoperative functional recovery. Additionally, linear regression analysis was used to examine correlations between nutritional status, PMA, and HOOS‐JR scores at the final follow‐up, incorporating Pearson's correlation coefficient to assess the strength and direction of associations [2, 5, 19]. All statistical analyses were performed using JMP 17 software (SAS Institute, Cary, NC). A two‐tailed *p*‐value < 0.05 was considered statistically significant.

## RESULTS

### Preoperative factors influencing the achievement of PASS on EQ‐5D

Age (*B* = 0.113, odds ratio = 1.12, 95% CI 1.01–1.24, *p* = 0.046), osteosarcopenia (*B* = −0.386, odds ratio = 0.68, 95% CI 0.49–0.94, *p* = 0.031), and spinopelvic stiffness (ΔSS < 10°, *B* = ‐0.452, odds ratio = 0.66, 95% CI 0.56–0.78, *p* = 0.028) were independently associated with lower likelihood of achieving the PASS threshold on the EQ‐5D (Table 1).

**Table 1:** Comparison of the demographic variables and multivariate logistic regression analyses between patients who did and did not achieve a PASS for the EuroQol 5‐Dimension 3‐Level scale.

	Achieved PASS	Did not achieve PASS	*B*	SE	Wald Chi‐square	Exp (B)	95% CI	*p*‐Value
Number of hips (%)	161 (66)	83 (34)						
Age, years	72 (5, 53 to 93)	65 (4, 52 to 91)	0.113	0.051	4.861	1.12	1.01 to 1.24	**0.046**
Men, *n* (%)	27 (17)	15 (18)	−0.010	0.295	0.001	0.99	0.56 to 1.76	0.983
BMI, kg/m^2^	24 (3, 18 to 31)	25 (3, 18 to 35)	0.039	0.247	0.025	1.04	0.64 to 1.69	0.531
CCI ≥ 3, *n* (%)	5 (3)	6 (7)	−0.545	0.415	1.727	0.58	0.26 to 1.31	0.337
Osteosarcopenia, *n* (%)	11 (7)	17 (20)	−0.386	0.167	5.320	0.68	0.49 to 0.94	**0.031**
Pelvic incidence < 60°, *n* (%)	119 (74)	49 (59)	0.148	0.102	2.130	1.16	0.95 to 1.42	0.137
Spinopelvic mobility, *n* (%)								
<10°	17 (11)	27 (32)	−0.452	0.083	25.123	0.66	0.56 to 0.78	**0.028**
≥30°	21 (13)	15 (18)	−0.094	0.186	0.259	0.91	0.63 to 1.31	0.483

*Note*: Data are expressed as mean (standard deviation, range) or number of hips involved (%), as appropriate for the data type. Bold text indicates *p* < 0.05, s significant between‐group differences of variables in the logistic regression.

Abbreviations: BMI, body mass index; CCI, Charlson Comorbidity Index; CI, confidence interval; EQ‐5D, European Quality of Life 5‐Dimension 3‐Level; PASS, patient‐acceptable symptom state; SE, standard error.

### Propensity‐score‐matched comparison and relationship between baseline variables and outcomes

After propensity‐score‐matching of patients who did (*n* = 35) and did not (*n* = 70) have preoperative spinopelvic mobility < 10°, all standardised mean differences were below 0.10, confirming the successful balancing of covariates between groups (Table 2). There were significant differences in PNI (*p* = 0.008), bone density and muscle status (*p* = 0.023), and MCID (*p* = 0.008 for EQ‐5D, and *p* < 0.001 for both HOOS‐JR and low back pain), as well as in PASS achievement (*p* < 0.001 for both EQ‐5D and HOOS‐JR; Table 3). Although no significant differences were observed in dislocation rates (*p* = 0.155), the osteosarcopenia group had a higher incidence of periprosthetic fractures compared to other groups (*p* = 0.044).

**Table 2:** Comparison of the demographic characteristics between individuals who did and did not have preoperative spinopelvic mobility < 10°.

	All subjects		*p*‐Value	Propensity score‐matched		*p*‐Value	SMD
	Study group	Control group		Study group	Control group		
Number of hips (%)	38 (18)	170 (82)		35 (33)	70 (67)		
Age, years	75 (5, 52–90)	68 (5, 55–93)	**0.011**	74 (7, 52–88)	71 (6, 58–93)	0.127	0.07
Men, *n* (%)	5 (13)	31 (18)	0.455	5 (14)	12 (17)	0.708	0.04
BMI, kg/m^2^	24 (4, 20–32)	24 (4, 18–35)	0.885	24 (4, 18–30)	23 (4, 20–30)	0.999	0.01
CCI ≥ 3, *n* (%)	5 (13)	4 (2)	**0.003**	4 (11)	2 (3)	0.075	0.09
EQ‐5D	0.41 (0.17–0.48)	0.43 (0.16–0.52)	0.643	0.41 (0.17–0.46)	0.42 (0.17–0.51)	0.776	0.03
HOOS‐JR	44.2 (5.2–31 to 53)	45.4 (5,1 34–55)	0.424	44.8 (5.1, 34–51)	45.1 (5.0, 35–53)	0.675	0.05
Follow‐up duration, years	2.3 (2.1–3.3)	2.3 (2.1–3.1)	0.762	2.3 (2.1–3.2)	2.3 (2.2–2.8)	0.857	0.02

*Note*: Data are expressed as mean (standard deviation, range) for normally distributed variables, median value (interquartile range) for variables not normally distributed, or number of hip involvements (%), as appropriate for the data type. Bold text indicates *p* < 0.05, significant between‐group differences.

Abbreviations: BMI, body mass index; CCI, Charlson Comorbidity Index; EQ‐5D, EuroQol 5‐Dimension 3‐Level scale; HOOS‐JR, Hip Disability and Osteoarthritis Outcome Score Joint Replacement; SMD, standardised mean difference.

**Table 3:** Propensity score‐matched comparison of variables between individuals who did and did not have preoperative spinopelvic mobility < 10°.

	Propensity score‐matched		*p*‐Value
	Study group	Control group	
Number of hips (%)	35 (33)	70 (67)	
PNI ≤ 40, *n* (%)	6 (17)	3 (3)	**0.008**
Bone density and muscle status, *n* (%)			**0.023**
Normal	5 (14)	29 (41)	
Low psoas muscle area alone	12 (34)	22 (31)	
Low bone density alone	10 (29)	12 (17)	
Osteosarcopenia	8 (23)	7 (10)	
Achieved MCID, *n* (%)			
EQ‐5D > 0.27	18 (52)	54 (77)	**0.008**
HOOS‐JR > 18	21 (60)	65 (92)	**<0.001**
VAS‐hip pain > 18.6 mm	34 (96)	70 (100)	0.155
VAS‐low back pain > 20 mm	18 (52)	68 (96)	**<0.001**
Achieved PASS, *n* (%)			
EQ‐5D > 0.77	15 (44)	53 (75)	**<0.001**
HOOS‐JR > 77	14 (40)	62 (88)	**<0.001**
Patient satisfaction, *n* (%)^a^	20 (56)	68 (96)	**0.005**
Postoperative dislocation, *n* (%)	1 (3)	0	0.155
Periprosthetic fracture, *n* (%)	2 (6)	0	**0.044**
Final follow‐up			
SS sitting, °	33 (7)	22 (10)	**0.012**
SS standing, °	37 (6)	41 (10)	0.601
ΔSS < 10°, *n* (%)	30 (86)	21 (30)	**<0.001**
Changes from baseline			
SS sitting, °	3 (7)	−1 (8)	0.763
SS standing, °	1 (8)	−2 (8)	0.301
ΔSS, °	2 (4)	0 (4)	0.562

*Note*: Data are expressed as mean (standard deviation) or number of hips involved (%), as appropriate for the data type. Bold text indicates *p* < 0.05, significant between‐group differences.

Abbreviations: EQ‐5D, EuroQol 5‐Dimension 3‐Level scale; HOOS‐JR, Hip Disability and Osteoarthritis Outcome Score Joint Replacement; MCID, minimum clinically important difference; PASS, patient‐acceptable symptom state; PNI, prognostic nutritional index = 10 × serum albumin (g/dL) + 0.005 × total lymphocyte count (/mm^3^); SS, sacral slope; VAS, visual analogue scale; Δ, difference between the standing and sitting values.

^a^ Count (%) of patients who reported being satisfied or very satisfied, graded on a 5‐point scale.

**Figure 4:**
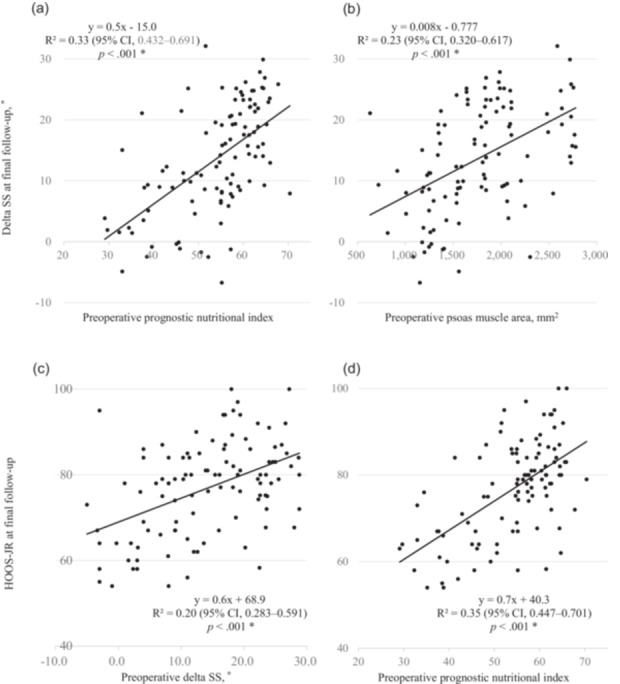
Correlation analyses of spinopelvic mobility, nutritional/muscle indices, and functional outcomes. (a) Postoperative spinopelvic mobility (ΔSS) shows a moderate positive correlation with preoperative Prognostic Nutritional Index (PNI) (*R* = 0.58, *p* < 0.001), suggesting that better nutritional status may support greater pelvic adaptability. (b) ΔSS is also positively correlated with preoperative psoas muscle area (PMA) (*R* = 0.48, *p* < 0.001), indicating a link between muscle mass and spinopelvic flexibility. (c) Preoperative ΔSS is positively associated with HOOS‐JR scores at final follow‐up (≥24 months postoperatively; *R* = 0.45, *p* = 0.028), suggesting that greater mobility prior to surgery may contribute to better postoperative function. (d) PNI also correlates positively with HOOS‐JR (*R* = 0.59, *p* < 0.001), supporting its potential utility as a non‐radiographic marker of musculoskeletal reserve and functional recovery. All panels include regression lines, *R*² values, and correlation coefficients. *p < 0.05 indicates statistical significance.

In the combined cohort (*n* = 105), both PNI (Figure 4a) and PMA (Figure 4b) showed positive correlations with postoperative ΔSS (*p* < 0.001). Additionally, preoperative ΔSS (Figure 4c) and PNI (Figure 4d) were positively associated with HOOS‐JR scores at final follow‐up (*p* < 0.001). Correlation coefficients and significance levels are summarised in tabular form (Supporting Information: Table S1). The change in ΔSS from baseline to final follow‐up was significantly associated with HOOS‐JR scores—positively in the study group and negatively in the control group, both reaching statistical significance (*p* < 0.05). However, when analysed in the combined cohort, the association did not remain significant (Supporting Information: Figure S1).

## DISCUSSION

This study demonstrated that both preoperative spinopelvic stiffness (ΔSS < 10°) and osteosarcopenia independently predict poor functional recovery following THA, highlighting the importance of incorporating biomechanical and musculoskeletal assessments into preoperative risk stratification. Interestingly, although older age is typically associated with reduced physiological reserves and higher comorbidity burdens, the results showed that increasing age was positively associated with PASS achievement. This counterintuitive finding may be explained by differences in patient expectations or preoperative functional baselines. Older adults may have more modest expectations or derive greater satisfaction from functional improvements that restore independence in basic activities of daily living. Similar trends have been reported in prior studies, including the external validation study by Florissi et al., which found that older age did not negatively impact PASS rates one year after THA [6]. These observations suggest that chronological age alone may not be a reliable predictor of patient satisfaction and underscore the importance of individualised preoperative counselling. Notably, the correlation between PNI and spinopelvic mobility suggests that PNI may serve as a non‐radiographic screening tool for identifying high‐risk patients, particularly in resource‐limited settings. PNI, a simple blood‐derived marker of nutritional and immunological status, showed significant correlations with both postoperative ΔSS and HOOS‐JR scores (Figure 4). Its accessibility and predictive utility make it a promising alternative to imaging‐based muscle mass assessments, particularly in preoperative screening. Our findings are consistent with previous studies indicating that restricted spinopelvic mobility negatively affects THA outcomes [12, 21]. The dynamic interaction between the pelvis and spine during postural transitions plays a crucial role in acetabular component orientation, joint stability, and functional recovery. The ΔSS < 10° threshold, widely used in biomechanical research, proved to be a clinically relevant predictor of postoperative recovery [9]. Given the increasing emphasis on preoperative spinopelvic evaluation in THA planning, these results further reinforce its role in optimising surgical decision‐making.

Osteosarcopenia, characterised by concurrent deficits in bone density and muscle mass, also emerged as a key determinant of suboptimal postoperative function [13, 17, 22]. This condition has been linked to impaired postural stability, reduced rehabilitation potential, and an increased risk of falls [17]. The ability to simultaneously assess both bone and muscle quality enhances perioperative risk stratification, particularly in orthopaedic populations where lower extremity muscle integrity is critical for postoperative function. This study also highlights a previously underexplored interplay between spinopelvic biomechanics and musculoskeletal health. While spinopelvic stiffness and osteosarcopenia independently influenced outcomes, their potential synergistic effects warrant further investigation. In the spinopelvic stiffness group, notably, higher preoperative SS in both sitting and standing positions were associated with poorer HOOS‐JR outcomes (Supporting Information: Table S1). This may reflect a biomechanical disadvantage in which excessive pelvic tilt, combined with restricted spinopelvic mobility, impairs dynamic sagittal balance during daily activities. In such cases, a fixed anterior pelvic tilt may lead to increased lumbar lordosis and elevated joint reaction forces across the hip and spine. This rigidity likely compromises the body's capacity to redistribute load efficiently during movement, resulting in suboptimal postoperative recovery. In contrast, patients with preserved spinopelvic mobility (the control group) may retain the ability to adjust pelvic orientation dynamically, thus mitigating the functional consequences of greater SS angles. We hypothesised that reduced spinopelvic mobility would impair the biomechanical adaptation necessary for postural transitions and load distribution after THA, while osteosarcopenia would limit the musculoskeletal capacity to compensate for such stiffness [23]. Their coexistence may, therefore, result in compounded functional impairment, contributing to inferior postoperative outcomes. A combined assessment of these factors may provide a more comprehensive framework for risk stratification. Future studies should examine whether targeted interventions, such as resistance training, nutritional optimisation, and core stabilisation, can mitigate the adverse effects of both spinopelvic dysfunction and osteosarcopenia, ultimately enhancing THA recovery.

The relationship between preoperative spinopelvic mobility and postoperative outcomes has been well‐documented, yet its predictive specificity remains a challenge [11, 12, 21, 23]. Our findings suggest that while preoperative ΔSS measurements provide valuable insights, incorporating musculoskeletal health markers, such as muscle mass and bone quality, may enhance risk stratification for THA outcomes (Figure 4). Importantly, this study identified PNI as a potential surrogate marker for spinopelvic mobility and postoperative recovery. This rationale is grounded in the concept that musculoskeletal health may influence spinopelvic function itself. Poor muscle quality could limit active pelvic mobility, while osteopenic bone may contribute to spinal rigidity due to degenerative changes. Thus, combining spinopelvic mobility with osteosarcopenia parameters is not merely additive but may reflect a mechanistically interrelated decline in spinopelvic biomechanics and structural musculoskeletal integrity. Similar to PMA, an established indicator of sarcopenia, PNI correlated with postoperative ΔSS and functional outcomes [19]. However, unlike PMA, which requires CT imaging and radiation exposure, PNI is a simple, widely available blood‐based biomarker, making it a more practical tool for preoperative risk assessment, particularly in settings where CT is not feasible. Given its accessibility and predictive potential, future studies should explore whether integrating nutritional indices into preoperative screening improves risk stratification and postoperative recovery.

This study has several limitations. First, its retrospective design introduces potential selection bias, although propensity score matching was employed to minimise confounding. No radiological data regarding cup positioning or the influence of other implants in the context of spinopelvic dysfunctions were reported [11, 12]. It is possible that in patients with limited spinopelvic mobility or hypermobility, different implants were chosen, or cup positioning was specifically adjusted, which may have had a significant impact on the outcome, only a very small proportion of patients (2.5%) received dual mobility implants, making it unlikely that implant heterogeneity introduced significant bias in our primary findings. Second, although postoperative ΔSS was calculated using SS values measured at the final follow‐up, longitudinal changes in spinopelvic biomechanics, such as during the early postoperative phase, were not assessed. THA has been reported to increase spinopelvic mobility by alleviating hip contractures and reducing compensatory pelvic tilt. The magnitude and clinical significance of such changes remain unclear, particularly in patients with preoperative stiffness or osteosarcopenia. Future studies incorporating serial radiographic and functional mobility assessments are needed to better understand these dynamics. Third, although the sample size was adequate for statistical analyses, larger prospective studies are warranted to validate these findings and refine predictive models by integrating additional musculoskeletal and biomechanical parameters. Moreover, the interaction between spinopelvic mobility and osteosarcopenia remains an underexplored determinant of THA outcomes. Fourth, muscle measurements were quantitative only; parameters such as fatty degeneration and muscle composition were not assessed, which may influence muscle function [5]. In considering spinopelvic mobility, the SS is provided as a parameter for spinopelvic mobility with threshold values. However, pelvic tilt is the commonly used parameter for which the characteristics between stiff and hypermobile are applied, even though the SS is directly related to pelvic tilt [9]. More research should evaluate whether a composite risk model incorporating both factors enhances predictive accuracy and whether targeted interventions—such as resistance training and perioperative nutritional optimisation—can improve postoperative recovery. Fifth, although baseline spinopelvic stiffness was predictive of poorer outcomes, the postoperative change in spinopelvic mobility did not consistently correlate with HOOS‐JR scores across all patients. While subgroup analyses showed opposing trends—with a significant negative association in the stiffness group and a positive one in the control group—these effects did not persist when evaluating the entire cohort. This suggests that isolated changes in ΔSS may not fully explain functional recovery and should be interpreted in conjunction with musculoskeletal conditions and patient‐specific factors such as activity level or expectations. Finally, although CT imaging was part of routine preoperative planning at our institution and did not involve exposure for research purposes, we acknowledge that this practice may not be universally standard. In some healthcare settings, the routine use of CT may be limited by concerns regarding radiation exposure or resource availability. While the explanatory power of the regression models was modest, this is not unexpected given the multifactorial nature of patient‐reported outcomes following THA. Functional recovery is shaped by diverse biomechanical and psychological influences that are difficult to fully quantify. Nevertheless, the identified predictors—spinopelvic stiffness and osteosarcopenia—are clinically meaningful due to their modifiability and potential to guide targeted preoperative interventions.

This study provided valuable insights into the relationship between spinopelvic mobility and osteosarcopenia on THA outcomes. By integrating validated imaging metrics and alternative tools, such as PNI, into clinical practice, clinicians can enhance preoperative risk stratification and optimise perioperative care for patients undergoing THA [2, 19]. The impact of abnormal spinopelvic mobility on postoperative recovery, particularly in patients with osteosarcopenia, remains a crucial area for future research. Understanding how changes in SS and other spinopelvic parameters relate to musculoskeletal health could help to optimise preoperative planning and surgical strategies, ultimately improving functional recovery and patient satisfaction. These efforts are crucial for managing ageing populations, where osteosarcopenia is increasingly prevalent, for improving surgical outcomes, recovery, and patient satisfaction.

## CONCLUSIONS

Preoperative spinopelvic stiffness and osteosarcopenia independently predict suboptimal functional recovery following THA. These findings emphasise the importance of comprehensive preoperative assessments incorporating both biomechanical and musculoskeletal health evaluations. The use of CT‐derived PMA and lumbar bone mineral density as alternative markers for muscle and bone quality provides a practical approach to risk stratification. Future research should focus on optimising predictive models and developing targeted interventions to improve THA outcomes in high‐risk populations.

## AUTHOR CONTRIBUTIONS

**Yoshinori Okamoto**: Conceptualisation; data curation; funding acquisition; writing–original draft; writing–review and editing. **Hitoshi Wakama**: Data curation; investigation. **Takafumi Saika**: Methodology; validation. **Kengo Tani**: Conceptualisation; data curation; investigation. **Shuhei Otsuki**: Validation; supervision.

## CONFLICT OF INTEREST STATEMENT

The authors declare no conflict of interest.

## ETHICS STATEMENT

The study was conducted in accordance with the ethical standards of the institutional and national research committees and with the 1964 Helsinki Declaration and its later amendments. The procedures used in this study were approved by our institutional ethics review board (approval number‐ 2020‐098‐1). All patients provided informed consent for the use of their clinical data for research purposes.

## Supporting information


**Supplementary Fig.** Scatter plot showing the relationship between changes in spinopelvic mobility (ΔSS) from preoperative to final follow‐up and HOOS‐JR scores at the final follow‐up. While opposing trends were observed between the study and control groups, no consistent correlation was found across the full cohort (n = 105; R = −0.13, *p* = .193), suggesting that longitudinal ΔSS changes alone may not sufficiently explain postoperative functional outcomes. *P < .05 indicates statistical significance.

**Supplementary Table.** Summary of correlation analyses between sacral slope, nutritional/muscle indices, and Hip Disability and Osteoarthritis Outcome Score–Joint Replacement scores.

## Data Availability

The data that support the findings of this study are available from the corresponding author, Yoshinori Okamoto, upon reasonable request.
