# Supplementary material for: Association of spinopelvic mobility and osteosarcopenia with total hip arthroplasty outcomes
Source: J Exp Orthop. 2025 Aug 5;12(3):e70395. doi: 10.1002/jeo2.70395 (PMC12322688; doi:10.1002/jeo2.70395)
Supplement: Supplementary file 1 — Supplementary Fig. Scatter plot showing the relationship between changes in spinopelvic mobility (ΔSS) from preoperative to final follow‐up and HOOS‐JR scores at the final follow‐up. While opposing trends were observed between the study and control groups, no consistent correlation was found across the full cohort (n = 105; R = −0.13, p = .193), suggesting that longitudinal ΔSS changes alone may not sufficiently explain postoperative functional outcomes. *P < .05 indicates statistical significance. [file JEO2-12-e70395-s001.pptx]

## Slide 1
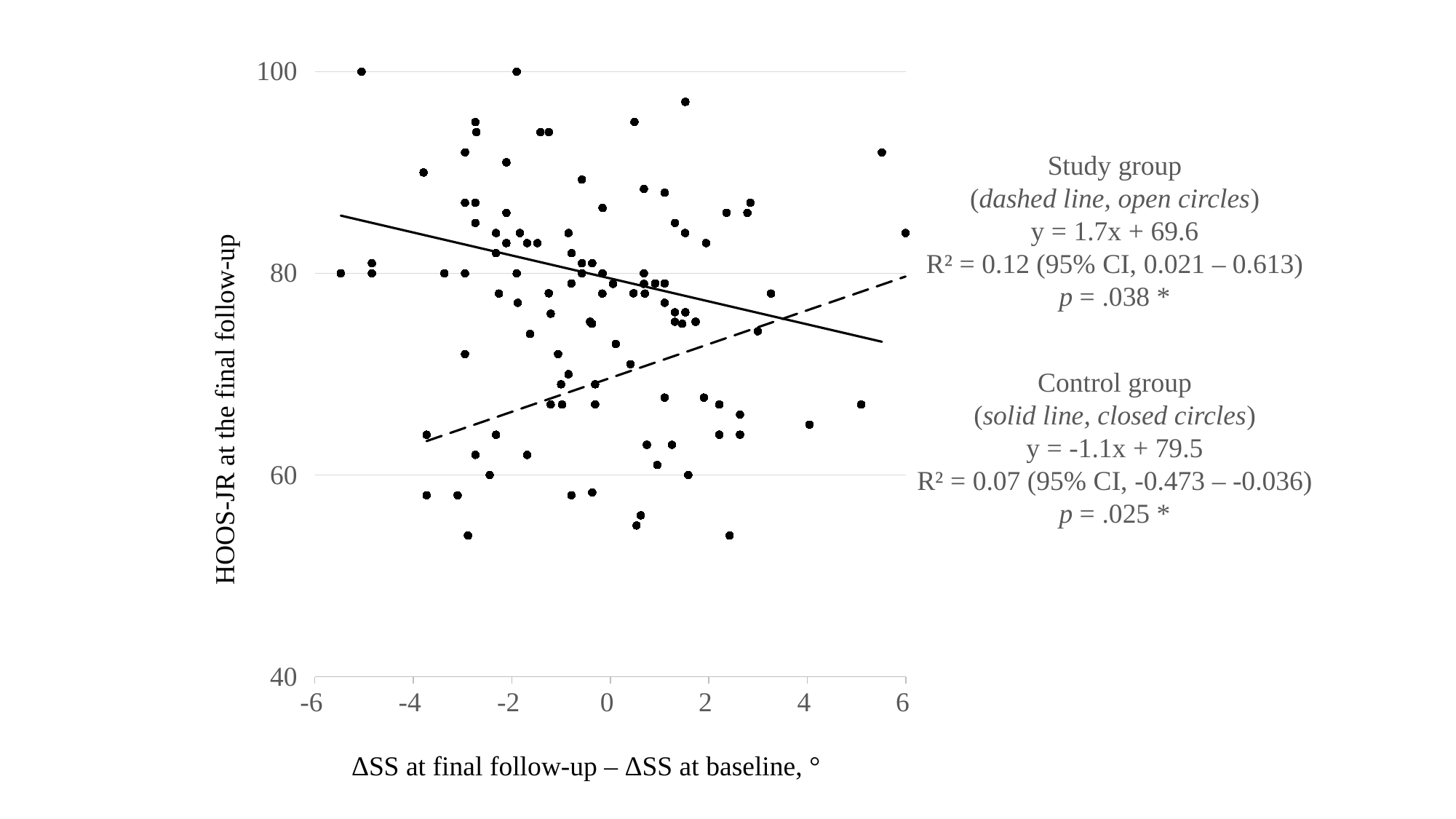

### Chart
| Category | C | S |
|---|---|---|Study group
(dashed line, open circles)
y = 1.7x + 69.6
R² = 0.12 (95% CI, 0.021 – 0.613)
p = .038 *
Control group
(solid line, closed circles)
y = -1.1x + 79.5
R² = 0.07 (95% CI, -0.473 – -0.036)
p = .025 *
HOOS-JR at the final follow-up
ΔSS at final follow-up – ΔSS at baseline, °
