# Supplementary material for: Association of spinopelvic mobility and osteosarcopenia with total hip arthroplasty outcomes
Source: J Exp Orthop. 2025 Aug 5;12(3):e70395. doi: 10.1002/jeo2.70395 (PMC12322688; doi:10.1002/jeo2.70395)
Supplement: Supplementary file 2 — Supplementary Table. Summary of correlation analyses between sacral slope, nutritional/muscle indices, and Hip Disability and Osteoarthritis Outcome Score–Joint Replacement scores. [file JEO2-12-e70395-s002.docx]

**Supplementary Table.** Summary of correlation analyses between sacral slope, nutritional/muscle indices, and Hip Disability and Osteoarthritis Outcome Score–Joint Replacement scores.

| Variable Pair (preoperative vs. final follow-up measure) | Group | Pearson R | 95% CI | *p* value | Number of hips |
| --- | --- | --- | --- | --- | --- |
| SS sitting vs. HOOS-JR | Study group | -0.63 | -0.79 to -0.37 | **<.001** | 35 |
|  | Control group | +0.35 | 0.12 to 0.54 | **.004** | 70 |
|  | Combined | -0.22 | -0.40 to -0.03 | **.024** | 105 |
| SS standing vs. HOOS-JR | Study group | -0.48 | -0.70 to -0.17 | **.004** | 35 |
|  | Control group | +0.46 | 0.25 to 0.63 | **<.001** | 70 |
|  | Combined | +0.32 | 0.13 to 0.48 | **.001** | 105 |
| Prognostic nutritional index vs. HOOS-JR | Study group | +0.52 | 0.22 to 0.72 | **.002** | 35 |
|  | Control group | +0.42 | 0.21 to 0.60 | **<.001** | 70 |
|  | Combined | +0.59 | 0.45 to 0.70 | **<.001** | 105 |
| Psoas muscle area vs. HOOS-JR | Study group | +0.59 | 0.32 to 0.77 | **<.001** | 35 |
|  | Control group | +0.59 | 0.40 to 0.72 | **<.001** | 70 |
|  | Combined | +0.67 | 0.55 to 0.77 | **<.001** | 105 |
| SS sitting vs. ΔSS | Study group | -0.25 | -0.53 to 0.09 | .147 | 35 |
|  | Control group | -0.39 | -0.58 to -0.17 | **<.001** | 70 |
|  | Combined | -0.53 | -0.66 to -0.38 | **<.001** | 105 |
| SS standing vs. ΔSS | Study group | +0.34 | 0.01 to 0.60 | **.046** | 35 |
|  | Control group | +0.55 | 0.36 to 0.70 | **<.001** | 70 |
|  | Combined | +0.67 | 0.55 to 0.77 | **<.001** | 105 |
| Prognostic nutritional index vs. ΔSS | Study group | +0.36 | 0.03 to 0.62 | **.035** | 35 |
|  | Control group | +0.53 | 0.33 to 0.68 | **<.001** | 70 |
|  | Combined | +0.58 | 0.51 to 0.76 | **<.001** | 105 |
| Psoas muscle area vs. ΔSS | Study group | +0.08 | -0.26 to 0.40 | .639 | 35 |
|  | Control group | +0.38 | 0.16 to 0.56 | **.001** | 70 |
|  | Combined | +0.48 | 0.37 to 0.65 | **<.001** | 105 |

Bold text indicates *P* < .05, s significant between-group differences of variables in the correlation analysis.

CI, confidence interval; HOOS-JR, Hip Disability and Osteoarthritis Outcome Score–Joint Replacement; SS, sacral slope; Δ, difference between the standing and sitting values.
